# Efficacy and safety of *Panax notoginseng* saponin injection in the treatment of acute myocardial infarction: a systematic review and meta-analysis of randomized controlled trials

**DOI:** 10.3389/fphar.2024.1353662

**Published:** 2024-03-21

**Authors:** Pengfei Chen, Zhuye Gao, Ming Guo, Deng Pan, He Zhang, Jianpeng Du, Dazhuo Shi

**Affiliations:** ^1^ Xiyuan Hospital, Cohina Academy of Chinese Medical Sciences, Beijing, China; ^2^ Cardiovascular Diseases Center, Xiyuan Hospital, China Academy of Chinese Medical Sciences, Beijing, China

**Keywords:** *Panax notoginseng* saponins, acute myocardial infarction, meta-analysis, systematic review, randomized controlled trials

## Abstract

**Purpose:** This study aimed to assess the efficacy and safety of *Panax notoginseng* saponin (PNS) injection, when combined with conventional treatment (CT), for acute myocardial infarction (AMI).

**Methods:** Comprehensive searches were conducted in seven databases from inception until 28 September 2023. The search aimed to identify relevant randomized controlled trials (RCTs) focusing on PNS injection in the context of AMI. This meta-analysis adhered to the PRISMA 2020 guidelines, and its protocol was registered with PROSPERO (number: CRD42023480131).

**Result:** Twenty RCTs involving 1,881 patients were included. The meta-analysis revealed that PNS injection, used adjunctively with CT, significantly improved treatment outcomes compared to CT alone, as evidenced by the following points: (1) enhanced total effective rate [OR = 3.09, *p* < 0.05]; (2) decreased incidence of major adverse cardiac events [OR = 0.32, *p* < 0.05]; (3) reduction in myocardial infarct size [MD = −6.53, *p* < 0.05]; (4) lower ST segment elevation amplitude [MD = −0.48, *p* < 0.05]; (5) mitigated myocardial injury as indicated by decreased levels of creatine kinase isoenzymes [MD = −11.19, *p* < 0.05], cardiac troponin T [MD = −3.01, *p* < 0.05], and cardiac troponin I [MD = −10.72, *p* < 0.05]; (6) enhanced cardiac function, reflected in improved brain natriuretic peptide [MD = −91.57, *p* < 0.05], left ventricular ejection fraction [MD = 5.91, *p* < 0.05], left ventricular end-diastolic dimension [MD = −3.08, *p* < 0.05], and cardiac output [MD = 0.53, *p* < 0.05]; (7) reduced inflammatory response, as shown by lower levels of C-reactive protein [MD = −2.99, *p* < 0.05], tumor necrosis factor-α [MD = −6.47, *p* < 0.05], interleukin-6 [MD = −24.46, *p* < 0.05], and pentraxin-3 [MD = −2.26, *p* < 0.05]; (8) improved vascular endothelial function, demonstrated by decreased endothelin-1 [MD = −20.56, *p* < 0.05] and increased nitric oxide [MD = 1.33, *p* < 0.05]; (9) alleviated oxidative stress, evidenced by increased superoxide dismutase levels [MD = 25.84, *p* < 0.05]; (10) no significant difference in adverse events [OR = 1.00, *p* = 1.00].

**Conclusion:** This study highlighted the efficacy and safety of adjunctive PNS injections in enhancing AMI patient outcomes beyond CT alone. Future RCTs need to solidify these findings through rigorous methods.

**Systematic Review Registration**: (https://www.crd.york.ac.uk/PROSPERO/), identifier (CRD42023480131)

## 1 Introduction

Acute myocardial infarction (AMI) represents irreversible myocardial damage, typically precipitated by the rupture of coronary artery plaque (Byrne et al., 2023). Annually, it is responsible for over 2.4 million fatalities in the United States, 4 million in Europe and northern Asia, and affects more than 7 million individuals worldwide (Yeh et al., 2010; Nichols et al., 2014). As a significant contributor to global disability and mortality, AMI poses a critical challenge in contemporary medicine (Reed et al., 2017). Treatment strategies for AMI prioritize the restoration of coronary flow to enable myocardial tissue reperfusion, primarily through percutaneous coronary intervention (PCI) and pharmacological thrombolysis. This approach is complemented by the administration of several medications. These include antiplatelet agents, antianginal agents, anticoagulants, and vasodilators. Vasodilators are also used to enhance cardiomyocyte nutrition. Additionally, beta-blockers and calcium antagonists are administered to reduce myocardial oxygen consumption (Montecucco et al., 2016; Reed et al., 2017; Sanchis et al., 2023). Although reperfusion alleviates ischemic myocardial injury, it can also cause specific, irreversible damage to cardiomyocytes (Gong et al., 2021; Murphy and Goldberg, 2022). Furthermore, existing treatments fall short in restoring the cardiac structure and function during prolonged AMI therapy, resulting in persistently high morbidity and mortality rates despite conventional drug therapy (Heusch and Gersh, 2017).

**FIGURE 1:**
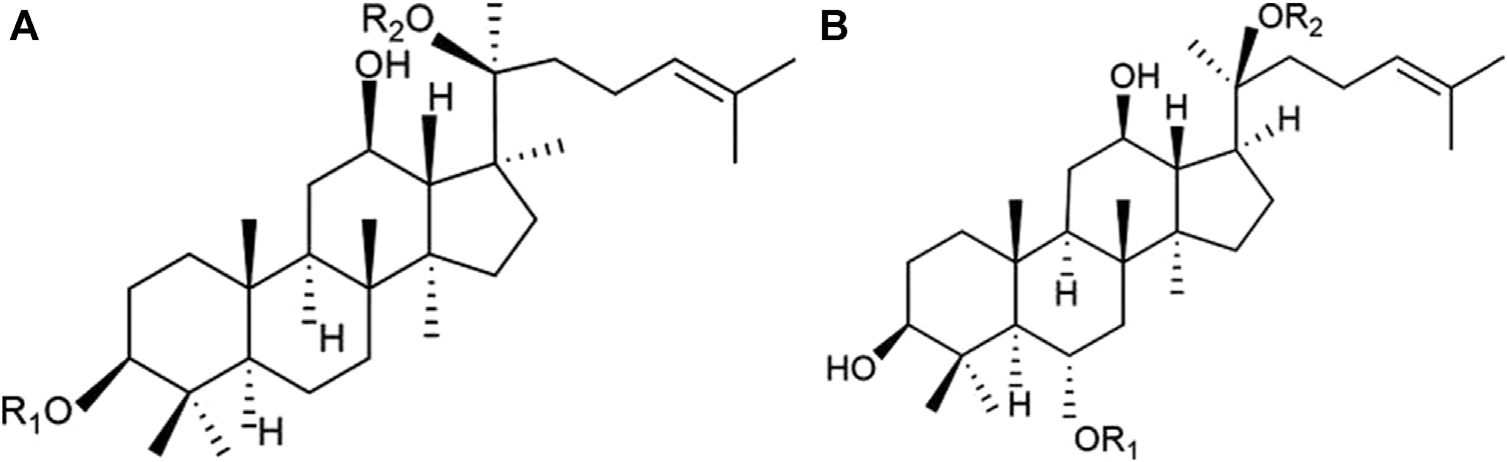
Chemical structures of the main saponins in PNS. **(A)** 20(S)-protopanaxatriol saponins, including notoginsenoside R1, ginsenoside Rg1, and Re, and **(B)** 20(S)- protopanaxadiol saponins, including ginsenoside Rb1 and Rd.

*Panax notoginseng* (PN), known as the root of *Panax notoginseng* (Burk*.*) F. H. Chen, is a botanical drug with an extensive history of medicinal use in traditional Chinese medicine (TCM) in China (Wang et al., 2016). Over centuries, PN has demonstrated significant efficacy in managing internal and external bleeding and ameliorating blood stasis, leading to its widespread use in clinical practice for the treatment of cardiovascular diseases (CVDs) (Liu et al., 2020). *Panax notoginseng* saponins (PNS), the freeze-dried extract of PN, comprises metabolites such as notoginsenoside R1 and ginsenosides Rb1, Rg1, Rd, and Re, as identified by high-performance liquid chromatography fingerprint analysis (Peng et al., 2018) (Figure 1) (Sun et al., 2020a). PNS formulations, including Xuesaitong, Xueshuantong , and Lulutong injections, have been clinically validated through trials and fundamental studies. These investigations substantiate PNS’s efficacy in reducing cardiac damage, mitigating inflammation, and inhibiting platelet aggregation (Zhou et al., 2018a; Wang et al., 2021a; Li et al., 2022; Wang et al., 2022; Zeng et al., 2023).

In recent years, there have been an increasing amount of randomized controlled trials (RCTs) using PNS injection to treat AMI. However, no comprehensive literature review or meta-analysis has assessed the efficacy and safety of these interventions. Consequently, this study aims to elucidate the cardioprotective effects of PNS injection in AMI patients.

## 2 Methods

### 2.1 Search strategy

Comprehensive searches were conducted in PubMed, EMBASE, Web of Science, Cochrane Library, Chinese National Knowledge Infrastructure, Wanfang, and VIP databases from inception until 28 September 2023. The search expression used in MEDLINE was (“acute myocardial infarction” OR “AMI” OR “st elevated myocardial infarction” OR “STEMI”) AND (“*panax notoginseng saponins*” OR “PNS” OR “sanqi” OR “sanchi” OR “xuesaitong” OR “xueshuantong”) AND (“randomized controlled trial” OR “RCT”). The detailed search strategies for all databases are presented in Supplementary Table S1. Titles, abstracts, and full texts were independently reviewed and cross-checked by two investigators (Pengfei Chen and Deng Pan), and any disagreement was decided by the senior reviewer (Dazhuo Shi).

### 2.2 Inclusion criteria

#### 2.2.1 Type of study

The study type was RCT, with no restrictions on languages, publication date, or publication status.

#### 2.2.2 Research object

All patients meeting any one of the current or previous diagnostic criteria for AMI were included. According to the Fourth Universal Definition of Myocardial Infarction (2018) standard (Thygesen et al., 2019), AMI is defined as acute myocardial injury [increased and/or decreased serum cardiac troponin (cTn), with at least once higher than the upper limit of the normal value (99 percentile of the upper limit of the reference value)], and there is at least one evidence of myocardial ischemia: (1) myocardial ischemic symptoms; (2) new ST-T changes or a new left bundle branch block (LBBB) observed in electrocardiogram (ECG); (3) a pathological Q-wave appeared in ECG; (4) imaging showed new loss of viable myocardium or new regional wall motion abnormalities; and (5) coronary angiography or autopsy confirmed thrombus in the coronary artery.

#### 2.2.3 Intervention

The control group (CG) received conventional treatment (CT), which was the first-line therapy regimens recommended in the current guideline. The experimental group (EG) received PNS injection plus CT, and the courses and dosages of PNS injection were not limited.

#### 2.2.4 Outcome indicators

The primary efficacy outcomes were total effective rates and major adverse cardiac events (MACEs). The secondary efficacy outcomes were (1) myocardial infarct size (MIS); (2) ST segment elevation amplitude; (3) myocardial injury markers: creatine kinase isoenzymes (CK-MB), cardiac troponin I (cTnI), and cardiac troponin T (cTnT); (4) cardiac function index: brain natriuretic peptide (BNP), left ventricular ejection fraction (LVEF), left ventricular end-diastolic dimension (LVEDD), and cardiac output (CO); (5) inflammatory response markers: C-reactive protein (CRP), tumor necrosis factor-α (TNF-α), interleukin-6 (IL-6), and pentraxin-3 (PTX-3); (6) vascular endothelial function: endothelin-1 (ET-1) and nitric oxide (NO); and (7) oxidative stress: superoxide dismutase (SOD). The safety outcome was adverse events.

### 2.3 Exclusion criteria

The exclusion criteria are as follows: (1) the intervention included other TCM (oral PNS, other Chinese medicine injections, and oral Chinese patent medicines) other than PNS injection; (2) research data had obvious deficiencies or serious errors; (3) patients with severe comorbidities such as severe heart failure, severe arrhythmia, and severe liver and renal dysfunction; and (4) duplicate publication.

### 2.4 Data extraction

The document information extraction table was established by Excel software, including basic characteristics of trials (first author, publication year, journal, and country), characteristics of patients (sample size, gender, age, and medical history), PNS injection and CT treatments (initial time, dosage, frequency, duration, and mean follow-up time), and outcome indicators.

### 2.5 Bias assessment

Two investigators (Pengfei Chen and Deng Pan) independently evaluated the quality assessment and risk of bias using the Cochrane Risk of Bias Tool 2.0, according to the following six aspects: (1) random sequence generation; (2) allocation concealment; (3) blinding (patients, medical staff, outcome evaluations, and data analysis); (4) incompletion of the outcome report (important indicators and follow-up rate); (5) selective reporting; and (6) other biases (such as baseline imbalance and suspected fraud. The two investigators’ answer of “yes” indicated a lower risk of bias, “no” indicated a higher risk of bias, and “unclear” indicated an uncertain risk of bias.

### 2.6 Statistical analysis

Statistical analysis was conducted using RevMan 5.4 software. Odds ratios (ORs) were used to represent categorical variables, and mean differences (MDs) were used for continuous variables while calculating 95% confidence intervals (CIs). The Cochrane’s Q test and the I^2^-value were used to assess the statistical heterogeneity, where *p* > 0.1 or I^2^ ≤ 50% suggested a favorable homogeneity among studies, and a fixed-effects model was used; *p* ≤ 0.1 or I^2^>50% suggested significant heterogeneity, so a random-effects model was used. When only two trials were included, although the heterogeneity was not significant, a random-effects model was selected to ensure the accuracy of the results. For sensitivity analysis, one study was omitted at a time to find the source of publication for heterogeneity, such as interventions, gender, age, and outcome indicators. When the number of trials was ≥10, we used the funnel chart to test whether there was publication bias. Although planned, we did not check the publication bias using Egger’s or Begg’s test as these tests were unreliable when fewer than 10 trials were included.

## 3 Results

### 3.1 Study search

**FIGURE 2:**
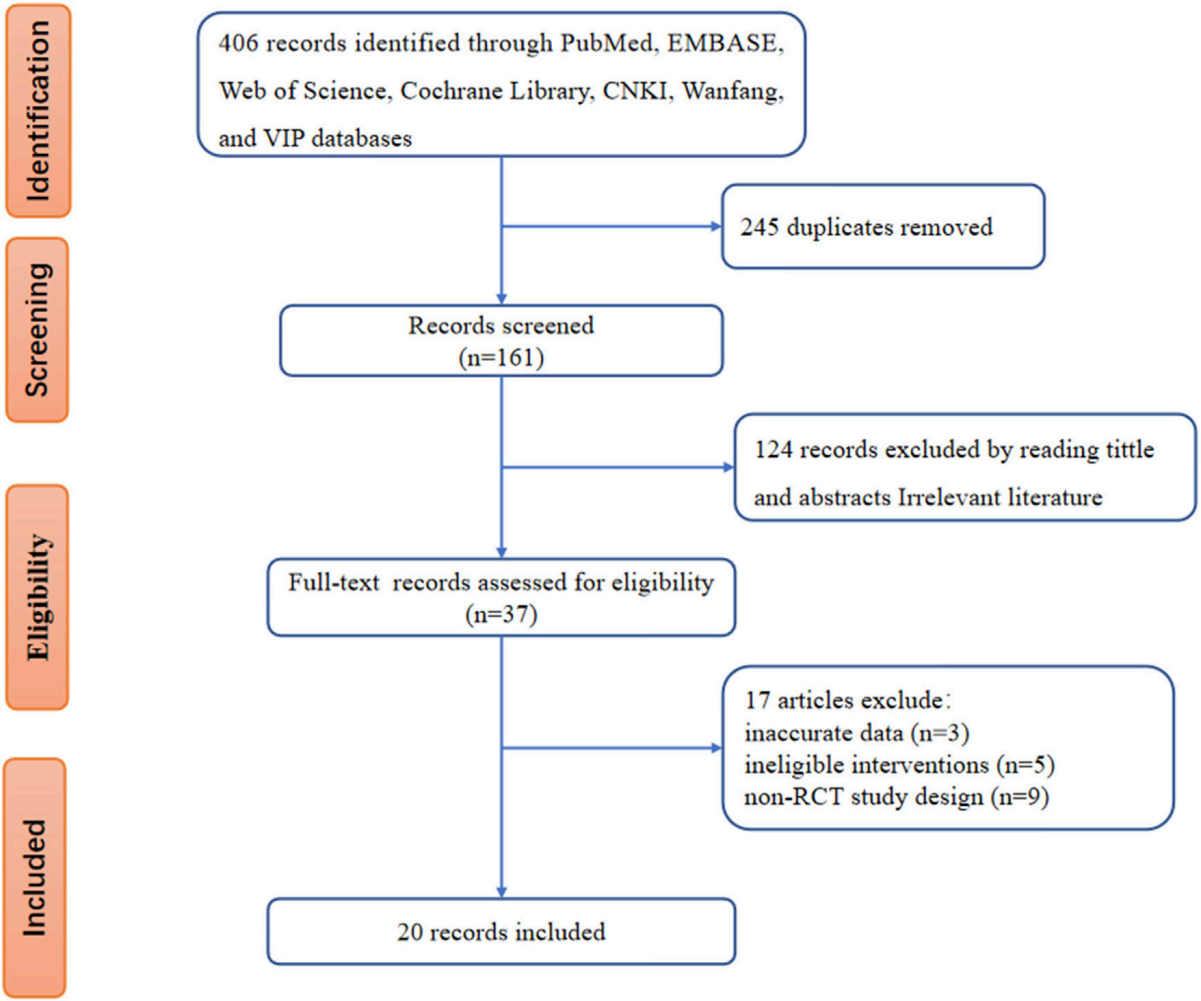
Flow chart of study selection and identification.

The process of the database search and study identification is presented in Figure 2. The initial database search identified 406 records. We excluded 245 duplicate records and 124 ineligible records by screening titles and abstracts. Of the remaining 37 articles that underwent full-text review, 17 were further excluded because of inaccurate data, ineligible interventions, and non-RCT study design. The exclusion studies and reasons are presented in Supplementary Table S2. Finally, 20 RCTs (Gan et al., 2010; Fu et al., 2014a; Fu et al., 2014b; Fan et al., 2015; Qiao et al., 2016; Feng et al., 2017; Xie, 2017; Liu et al., 2018; Wang, 2018; Xin and Qian, 2018; Zhou, 2018; Chen, 2019; Guo, 2019; Zhang, 2019; Zhang and Du, 2019; Zhong and Zheng, 2019; Sun et al., 2020b; Sun, 2021; Ji and Liu, 2022; Yang et al., 2022) were included.

### 3.2 Basic information

All 20 RCTs were published in China from 2010 to 2023. The total sample size was 1,881 cases, with a maximum of 138 cases and a minimum of 39 cases. In total, there were 943 cases in the EG and 938 cases in the CG. According to the ConPhyMP guidelines (Heinrich et al., 2022), all included RCTs focused on “type A” extracts (documented in the national pharmacopeia with licensed applications). The composition, source, and chemical characteristics of PNS injection used in the included trials are presented in Supplementary Table S3. The other details are shown in Table 1.

**TABLE 1:** Characteristics of included studies.

Author (publication year)	Sample size (T/C)	Male/female		Age (year)	Intervention		Reperfusion strategy	Courses/day	Follow-up/week	Outcomes
		T	C	T/C	T	C				
Yang et al. (2022)	55/55	35/20	31/24	62.45 ± 8.12/61.74 ± 7.69	Xueshuantong injection 300 mg + 5% G.S. 250 mL qd iv gtt	Aspirin, Plavix, low-molecular weight heparin, Tatin, ACEI, β-blockers, nitrates, and rhBNP	PCI	7 days	24w	⑧⑩⑱
Yang et al. (2015)	42/42	24/18	22/20	64.1 ± 9.2/62.7 ± 9.6	Xueshuantong injection 250 mg + 5% G.S. 250 mL qd iv gtt	Aspirin, Plavix, urokinase injection, and low-molecular weight heparin calcium	Thrombolysis	14 days	8w	④⑤⑥⑮⑰⑱
Zhong and Zheng. (2019)	69/69	38/31	36/33	51.7 ± 5.3/49.9 ± 6.1	Thrombolysis at the same time administering Xueshuantong injection 500 mg + 0.9% N.S. 250 mL qd iv gtt	Urokinase injection, aspirin, and alprostadil injection	Thrombolysis	7 days	1w	②⑧⑮⑯
Xie. (2017)	40/40	24/16	22/18	73.4 ± 6.3/72.6 ± 6.7	Xueshuantong injection 300 mg + 0.9% N.S. 250 mL qd iv gtt	CT	PCI	7 days	4w	①④⑥⑧⑨⑮⑰⑱
Guo. (2019)	44/44	27/17	25/19	55.28 ± 8.36/55.59 ± 8.14	Before PCI, Xuesaitong injection 400 mg iv drop. After PCI, Xuesaitong injection 400 mg + 0.9% N.S. 250 mL qd iv gtt	Aspirin, Plavix, low-molecular weight heparin, tirofiban hydrochloride, Tatin, ACEI, and β-blockers	PCI	14 days	4w	④⑤⑦⑧⑨
Sun et al. (2020a)	50/50	34/16	35/15	55.83 ± 8.25/55.94 ± 8.39	Before PCI, Xuesaitong injection 100 mg iv drop. After PCI, Xuesaitong injection 100 mg + 0.9% N.S. 250 mL qd iv gtt	Aspirin, Plavix, low-molecular weight heparin, tirofiban hydrochloride, Tatin, ACEI, and β-blockers	PCI	14 days	2w	⑧⑨⑪⑫⑲
Zhou et al. (2018a)	62/62	33/29	35/27	55.35 ± 7.24/54.79 ± 7.45	Xuesaitong injection 200 mg + 0.9% N.S. 250 mL qd iv gtt	Aspirin, Plavix, Tatin, ACEI, and β-blockers	PCI	14 days	4w	①④⑤⑦⑧⑨⑪⑫⑬⑭
Zhang. (2019)	60/60	38/22	41/19	51.13 ± 8.98/50.87 ± 7.94	Before PCI, Xuesaitong injection 100 mg iv drop. After PCI, Xuesaitong injection 100 mg + 0.9% N.S. 250 mL qd iv gtt	Aspirin, Plavix, and Tatin	PCI	14 days	2w	⑪⑫
Chen. (2019)	54/54	33/21	30/24	59.87 ± 4.31/59.79 ± 4.28	Before PCI, Xuesaitong injection 400 mg iv drop. After PCI, Xuesaitong injection 400 mg + 0.9% N.S. 250 mL qd iv gtt	Aspirin, Plavix, low-molecular weight heparin, and tirofiban hydrochloride	PCI	14 d	2w	④⑤⑦⑧⑨⑲
Qiao et al. (2016)	40/40	22/18	24/16	62.1 ± 7.9/63.5 ± 7.8	Xuesaitong injection 400 mg + 5% G.S. 250 mL qd iv gtt	Aspirin, Plavix, and Tatin	PCI	14 days	2w	⑪⑬
Sun et al. (2020b)	50/50	32/18	30/20	59.02 ± 6.15/59.14 ± 6.26	Xuesaitong injection 400 mg + 5% G.S. 250 mL qd iv gtt	Alteplase + CT	Thrombolysis	15 days	2w	①②⑧⑩
Ji and Liu. (2022)	54/54	33/21	35/19	53.42 ± 9.65/53.79 ± 9.46	Xueshuantong injection 500 mg + 5% G.S. 250 mL qd iv gtt	Alteplase, Low-molecular weight heparin, ACEI, β-blockers, and nitrates	Thrombolysis	7 days	4w	①④⑥⑧⑨⑱
Xin and Qian. (2018)	54/53	33/21	31/22	51.9 ± 8.4/52.3 ± 8.2	Before PCI, Xuesaitong injection 100 mg iv drop. After PCI, Xuesaitong injection 100 mg + 0.9% N.S. 250 mL qd iv gtt	Aspirin, Plavix, and Tatin	PCI	14 days	2w	⑪⑫⑱
Zhang and Du, (2019)	40/40	28/12	26/14	67.23 ± 3.31/66.84 ± 3.53	Xuesaitong injection 400 mg + 0.9% G.S. 250 mL qd iv gtt	Edaravone + CT	—	14 days	2w	⑧⑨⑪
Fu et al. (2014a)	30/30	—	—	—	Thrombolysis at the same time administering Xueshuantong injection 250 mg + 5% G.S. 250 mL qd iv gtt	Urokinase + CT	Thrombolysis	7 days	1w	⑦
Fu et al. (2014b)	36/32	20/16	17/15	52.6/53.8	Thrombolysis at the same time administering Xueshuantong injection 250 mg + 5% G.S. 250 mL qd iv gtt	Urokinase + CT	Thrombolysis	14 days	24w	②⑥⑧⑱
Liu et al. (2018)	60/60	36/24	37/23	57.66 ± 10.12/57.55 ± 10.09	Before PCI, Xuesaitong injection 400 mg iv drop. After PCI, Xuesaitong injection 400 mg + 0.9% N.S. 250 mL qd iv gtt	Aspirin, Plavix, low molecular weight heparin, tirofiban hydrochloride, Tatin, ACEI, and β-blockers	PCI	14 days	4w	④⑤⑦⑧⑨⑪⑭⑲
Wang. (2018)	52/52	31/21	28/24	56.71 ± 6.25/57.29 ± 6.61	Before PCI, Xuesaitong injection 400 mg iv drop. After PCI, Xuesaitong injection 400 mg + 0.9% N.S. 250 mL qd iv gtt	Aspirin, Plavix, low-molecular weight heparin, tirofiban hydrochloride, Tatin, and β-blockers	PCI	14 days	4w	⑧⑨⑲
Feng et al. (2017)	31/32	—	—	—	Xuesaitong injection 400 mg. Intracoronary injection via a guided catheter	Aspirin, Plavix, and low-molecular weight heparin	PCI	14 days	24w	③
Gan et al. (2010)	20/19	12/8	11/8	57.6 ± 10.2/55.4 ± 9.8	Xuesaitong injection 400 mg, intracoronary injection via a guided catheter. After PCI, Xuesaitong injection 400 mg + 0.9% N.S. 250 mL qd iv gtt	Aspirin, Plavix, and tirofiban hydrochloride	PCI	2 days	24w	③⑱

T/C, treatment group/control group; PCI, percutaneous coronary intervention; CT, conventional treatment; qd, once a day; iv gtt, intravenous guttae; ① total effective rate, ② myocardial infarct size, ③ ST segment elevation amplitude, ④ CK-MB, ⑤ cTnT, ⑥ cTnI, ⑦ BNP, ⑧ LVEF, ⑨ LVEDD, ⑩ CO, ⑪ CRP, ⑫ TNF-α, ⑬ IL-6, ⑭ PTX-3, ⑮ ET-1, ⑯ NO, ⑰ SOD, ⑱ major adverse cardiac events, and ⑲adverse response.

### 3.3 Literature quality evaluation

**FIGURE 3:**
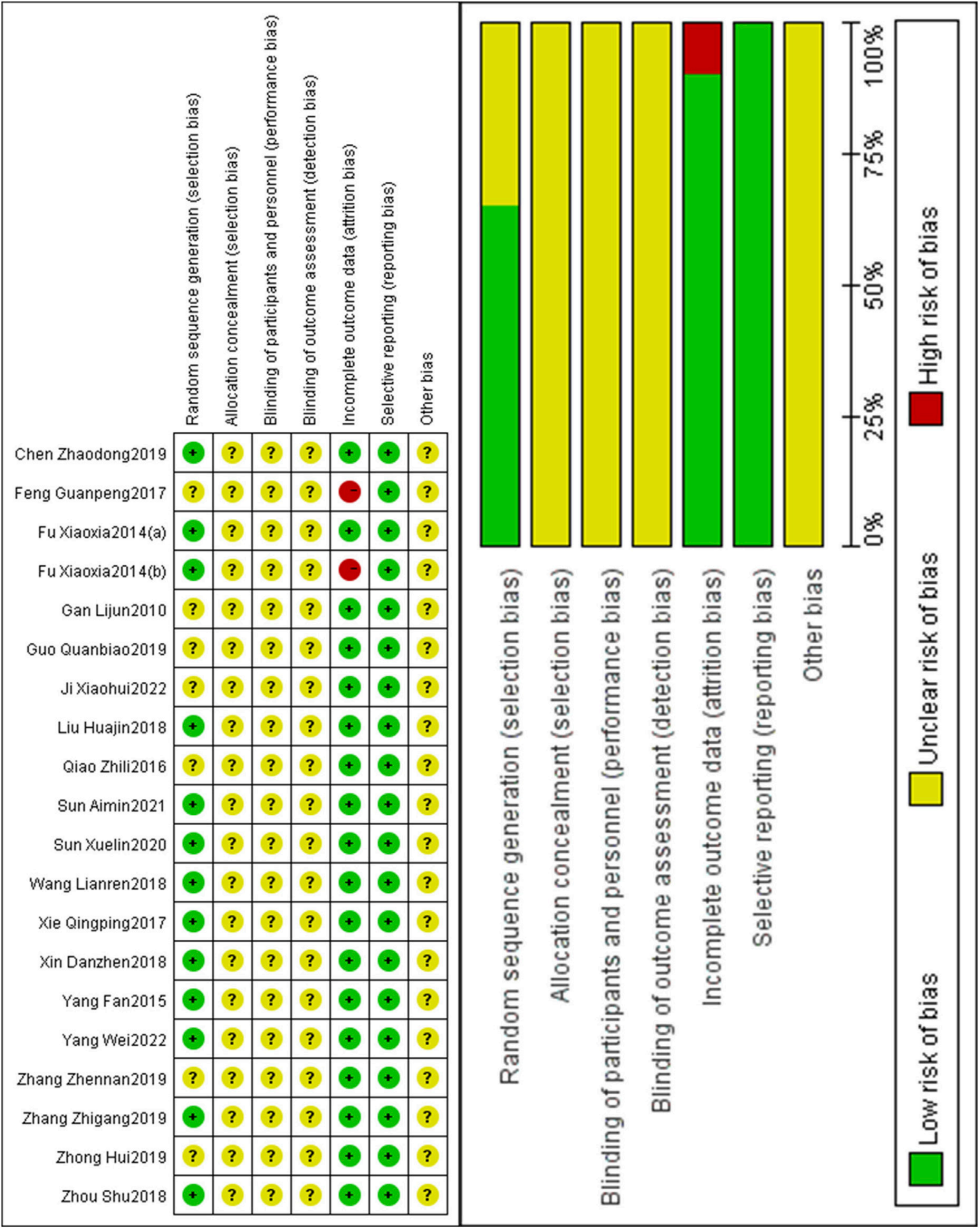
Risk of bias of included studies.

Regarding randomization, 13 RCTs (Fu et al., 2014a; Fu et al., 2014b; Fan et al., 2015; Xie, 2017; Liu et al., 2018; Wang, 2018; Xin and Qian, 2018; Zhou, 2018; Chen, 2019; Zhang, 2019; Sun et al., 2020b; Sun, 2021; Yang et al., 2022) generated random sequences using a random number table, which were rated as “low risk.” The remaining trials did not report the specific randomized method, resulting in an “unknown risk.” None of the RCTs reported the assignment hiding scheme, and the predictable future assignments could have led to the failure of randomization. Therefore, we rate the allocation concealment of all RCTs as “unknown risk”. Whether or not outcomes were measured by a blinded assessor could not be established because of the lack of information. Therefore, we rate the blinding of all RCTs as “unknown risk”. Two RCTs (Fu et al., 2014a; Feng et al., 2017) were rated as “high risk” due to incomplete data, which did not indicate the sample size and average age, while the others were rated as “low risk.” The results of all study reports were rated as “low risk” without selective reporting. No other bias was reported in any RCTs and was rated as “unknown risk” (Figure 3).

### 3.4 Main efficacy outcomes

#### 3.4.1 Total effective rate

**FIGURE 4:**
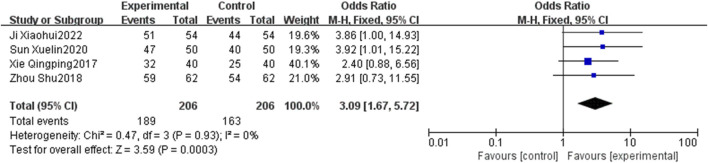
Forest plot of total effective rate.

Four RCTs (Xie, 2017; Zhou, 2018; Sun et al., 2020b; Ji and Liu, 2022) were reported on the total effective rate of PNS injection plus CT *versus* CT. The heterogeneity was not significant (*p* = 0.93, I^2^ = 0%), so a fixed-effects model was used. The results presented in Figure 4 show that the total effective rate in the EG is significantly higher than that in the CG, with statistically significant differences [OR = 3.09, 95% CI: 1.67 to 5.72, *p* < 0.05].

#### 3.4.2 MACEs

**FIGURE 5:**
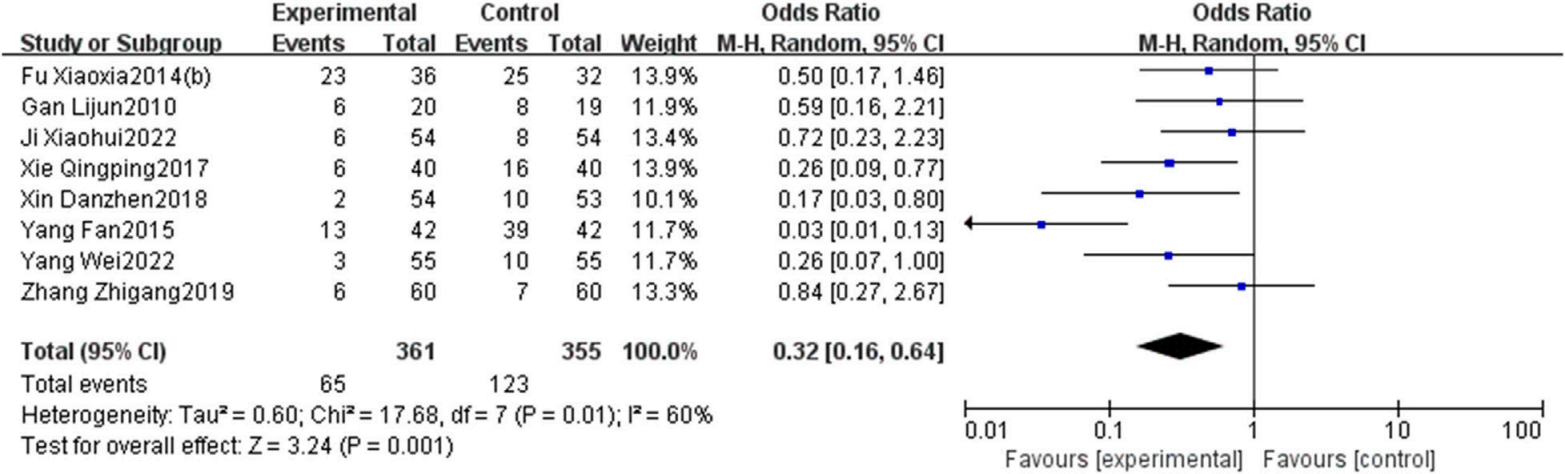
Forest plot of MACEs.

Eight RCTs (Gan et al., 2010; Fu et al., 2014a; Fu et al., 2014b; Fan et al., 2015; Xie, 2017; Xin and Qian, 2018; Zhang, 2019; Ji and Liu, 2022; Yang et al., 2022) investigated the incidence of MACEs. As the heterogeneity was significant (*p* = 0.01, I^2^ = 60%), a random-effects model was used. The results presented in Figure 5 show that the incidence of MACEs in the EG is significantly lower than that in the CG, and the differences were statistically significant [OR = 0.32, 95% CI: 0.16 to 0.64, *p* < 0.05].

### 3.5 Secondary efficacy outcomes

#### 3.5.1 MIS

**FIGURE 6:**
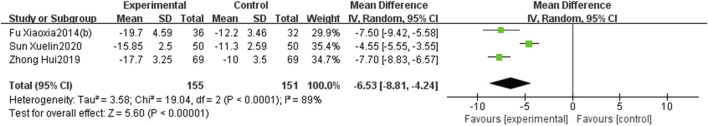
Forest plot of MIS.

Three RCTs (Fu et al., 2014b; Zhong and Zheng, 2019; Sun et al., 2020b) examined the MIS. The statistical heterogeneity among them was substantial (*p* < 0.01, I^2^ = 89%), so a random-effects model was used. As shown in the results, a statistical difference was found between the two groups, which meant that the MIS in the EG was significantly lower than that in the CG [MD = −6.53, 95% CI: −8.81 to −4.24, *p* < 0.05] (Figure 6).

#### 3.5.2 ST segment elevation amplitude

**FIGURE 7:**
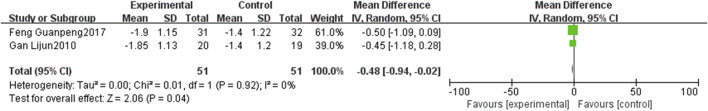
Forest plot of ST segment elevation amplitude.

Two RCTs (Gan et al., 2010; Feng et al., 2017) examined the ST segment elevation amplitude. Although the heterogeneity was not significant between the two studies (*p* = 0.92, I^2^ = 0%), the random-effects model was used. The results indicated that PNS injection plus CT significantly decreases the ST segment elevation amplitude compared with CT alone [MD = −0.48, 95% CI: −0.94 to −0.02, *p* < 0.05] (Figure 7).

#### 3.5.3 Myocardial injury

**FIGURE 8:**
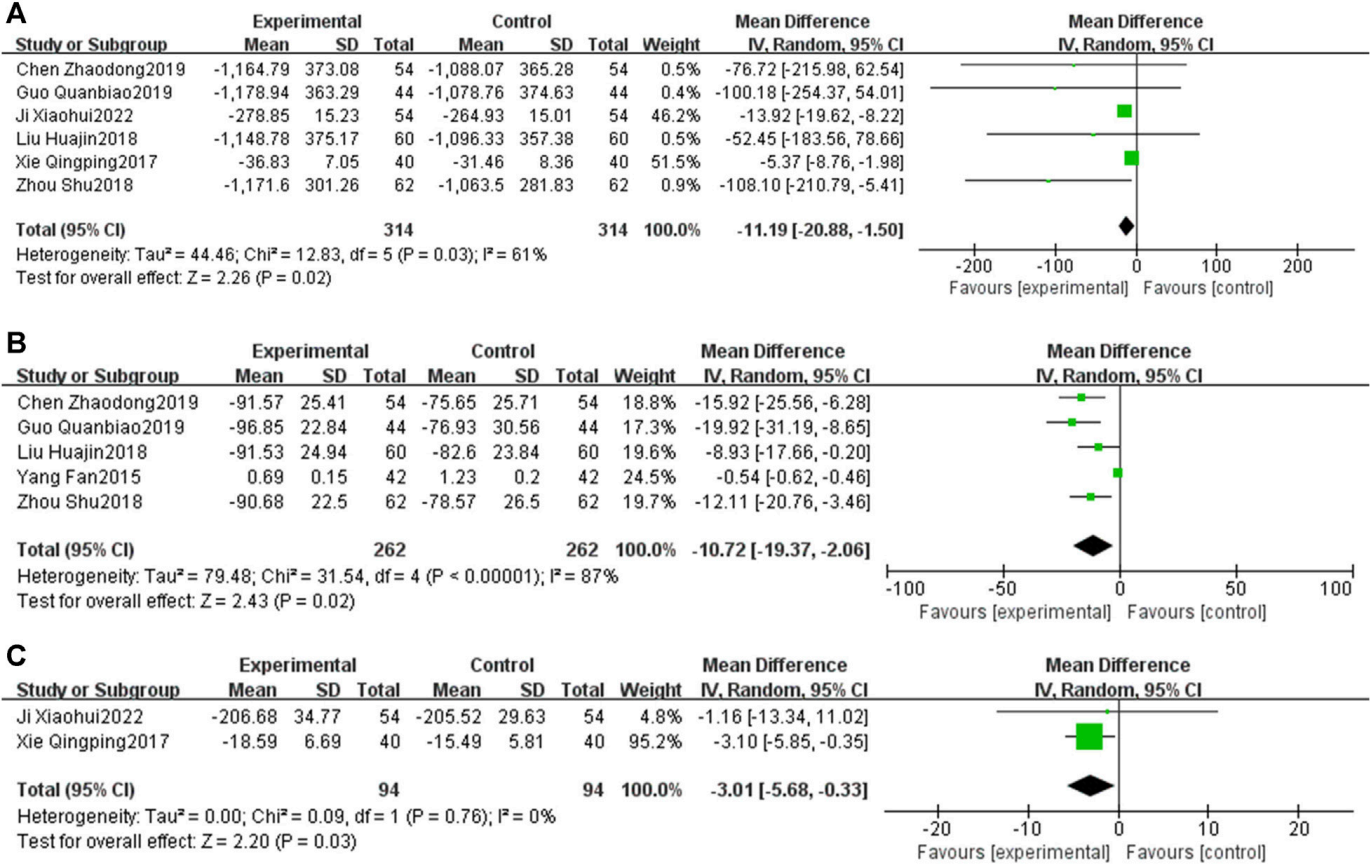
Forest plot of myocardial injury [**(A)** CK-MB; **(B)** cTnt; **(C)** cTnI].

Six RCTs (Xie, 2017; Liu et al., 2018; Zhou, 2018; Chen, 2019; Guo, 2019; Ji and Liu, 2022) reported on CK-MB. The meta-analysis revealed that the CK-MB levels in the EG were significantly lower than those in the CG [MD = −11.19, 95% CI: −20.88 to −1.50, *p* < 0.05; *I* ^2^ = 61%]. Five RCTs (Fan et al., 2015; Liu et al., 2018; Zhou, 2018; Chen, 2019; Guo, 2019) addressed the cTnT, with the meta-analysis indicating that cTnT levels in the EG were significantly reduced compared to those in the CG [MD = −10.72, 95% CI: −19.37 to −2.06, *p* < 0.05; *I* ^2^ = 87%]. Additionally, two RCTs (Xie, 2017; Ji and Liu, 2022) focused on cTnI, and the meta-analysis showed that cTnI levels in the EG were notably lower than those in the CG [MD = −3.01, 95% CI: −5.68 to −0.33, *p* < 0.05; *I* ^2^ = 0%] (Figure 8).

#### 3.5.4 Cardiac function

**FIGURE 9:**
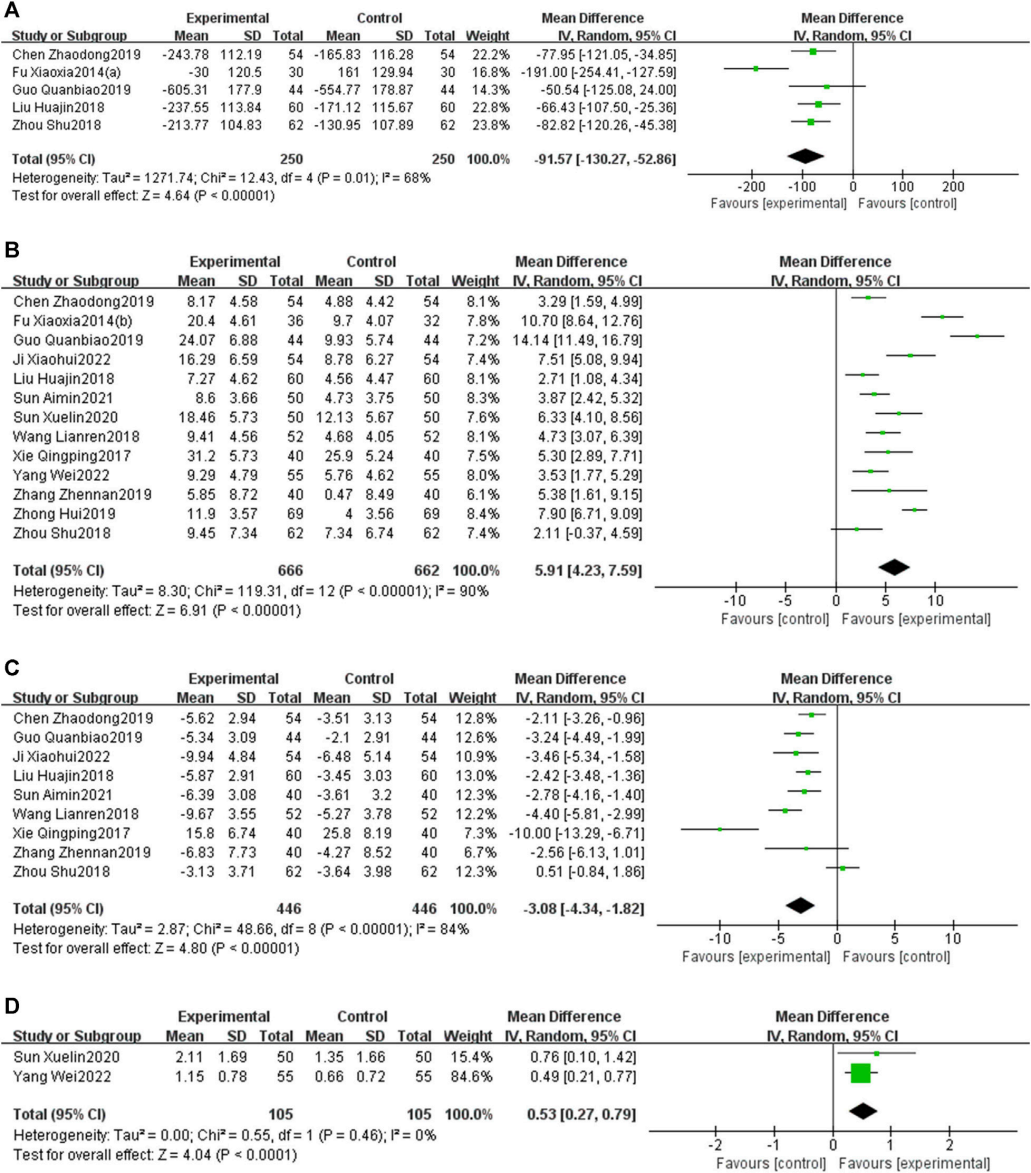
Forest plot of cardiac function [**(A)** BNP; **(B)** LVEF; **(C)** LVEDD; **(D)** CO].

Five RCTs (Fu et al., 2014a; Liu et al., 2018; Zhou, 2018; Chen, 2019; Guo, 2019) reported on BNP. The meta-analysis showed that the BNP in the EG is significantly lower than that in the CG [MD = −91.57, 95% CI: −130.27 to −52.86, *p* < 0.05; *I* ^2^ = 68%]. Thirteen RCTs (Fu et al., 2014b; Xie, 2017; Liu et al., 2018; Wang, 2018; Zhou, 2018; Chen, 2019; Guo, 2019; Zhang and Du, 2019; Zhong and Zheng, 2019; Sun et al., 2020b; Sun, 2021; Ji and Liu, 2022; Yang et al., 2022) focused on the LVEF, revealing that LVEF in the EG was significantly increased compared to that in the CG [MD = 5.91, 95% CI: 4.23 to 7.59, *p* < 0.05; *I* ^2^ = 90%]. Nine RCTs (Xie, 2017; Liu et al., 2018; Wang, 2018; Zhou, 2018; Chen, 2019; Guo, 2019; Zhang and Du, 2019; Sun, 2021; Ji and Liu, 2022) focused on the LVEDD, revealing that LVEDD in the EG was significantly reduced compared to that in the CG [MD = −3.08, 95% CI: −4.34 to −1.82, *p* < 0.05; *I* ^2^ = 84%]. Additionally, two RCTs (Sun et al., 2020b; Yang et al., 2022) reported on CO. Meta-analysis showed that the CO in EG is significantly higher than that in the CG [MD = 0.53, 95% CI: 0.27 to 0.79, *p* < 0.05; *I* ^2^ = 0%] (Figure 9).

#### 3.5.5 Inflammatory response

**FIGURE 10:**
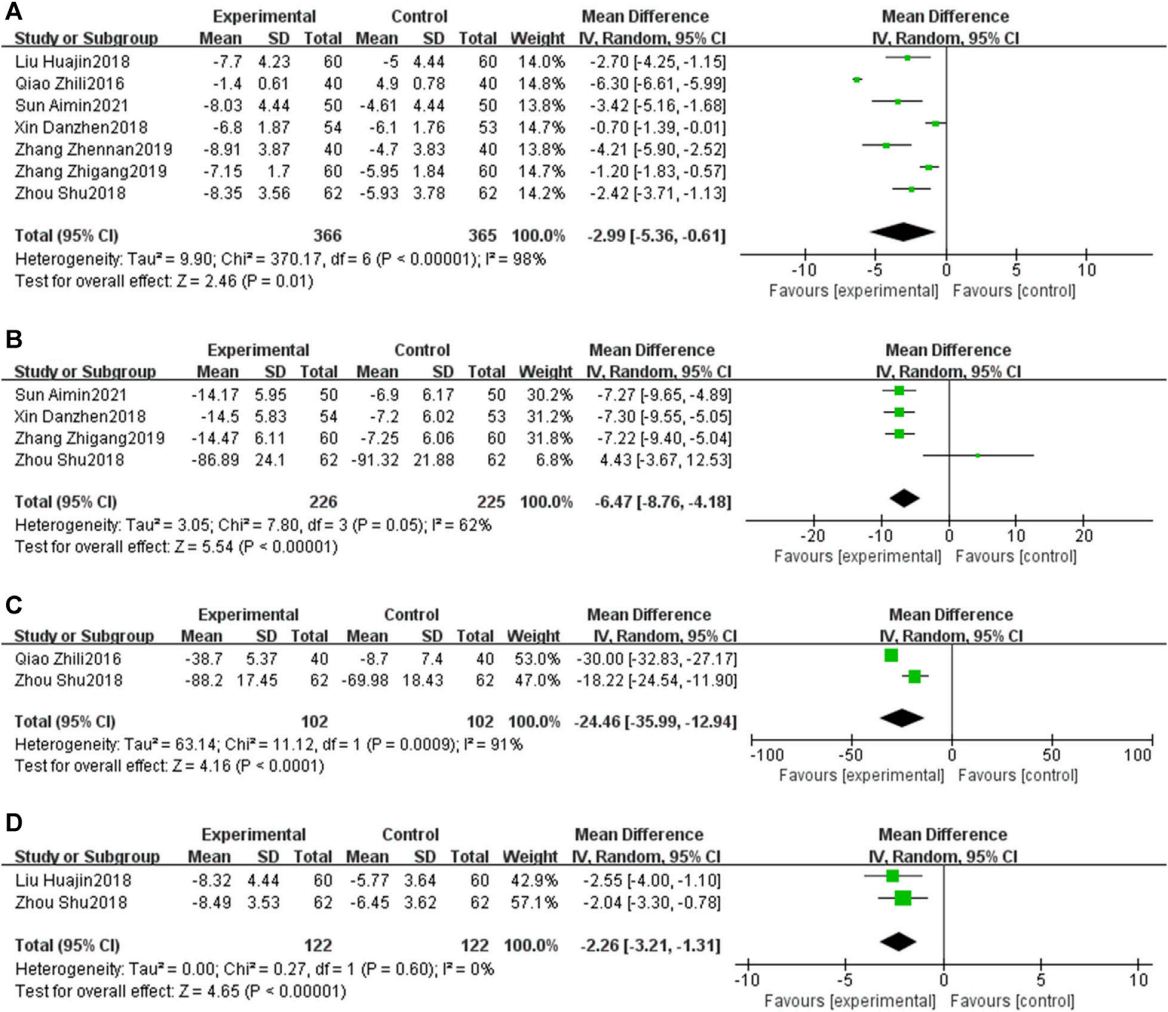
Forest plot of inflammatory response [**(A)** CRP; **(B)** TNF--α; **(C)** IL-6; **(D)** PTX-3].

Seven RCTs (Qiao et al., 2016; Liu et al., 2018; Xin and Qian, 2018; Zhou, 2018; Zhang, 2019; Zhang and Du, 2019; Sun, 2021) examined the CRP levels. The meta-analysis demonstrated that the CRP levels in the EG were significantly lower than those in the CG [MD = −2.99, 95% CI: −5.36 to −0.61, *p* < 0.05; *I* ^2^ = 98%]. Four RCTs (Xin and Qian, 2018; Zhou, 2018; Zhang, 2019; Sun, 2021) investigated TNF-α, revealing that TNF-α levels in the EG were significantly reduced compared to those in the CG [MD = −6.47, 95% CI: −8.76 to −4.18, *p* < 0.05; *I* ^2^ = 62%]. Two RCTs (Qiao et al., 2016; Zhou, 2018) reported on IL-6, and the meta-analysis indicated that IL-6 levels in the EG were significantly lower than in the CG [MD = −24.46, 95% CI: −35.99 to −12.94, *p* < 0.05; *I* ^2^ = 91%]. Similarly, two RCTs (Liu et al., 2018; Zhou, 2018) focused on PTX-3, and the meta-analysis showed that PTX-3 levels in the EG were significantly reduced compared to those in the CG [MD = −2.26, 95% CI: −3.21 to −1.31, *p* < 0.05; *I* ^2^ = 0%] (Figure 10).

#### 3.5.6 Vascular endothelial function

**FIGURE 11:**
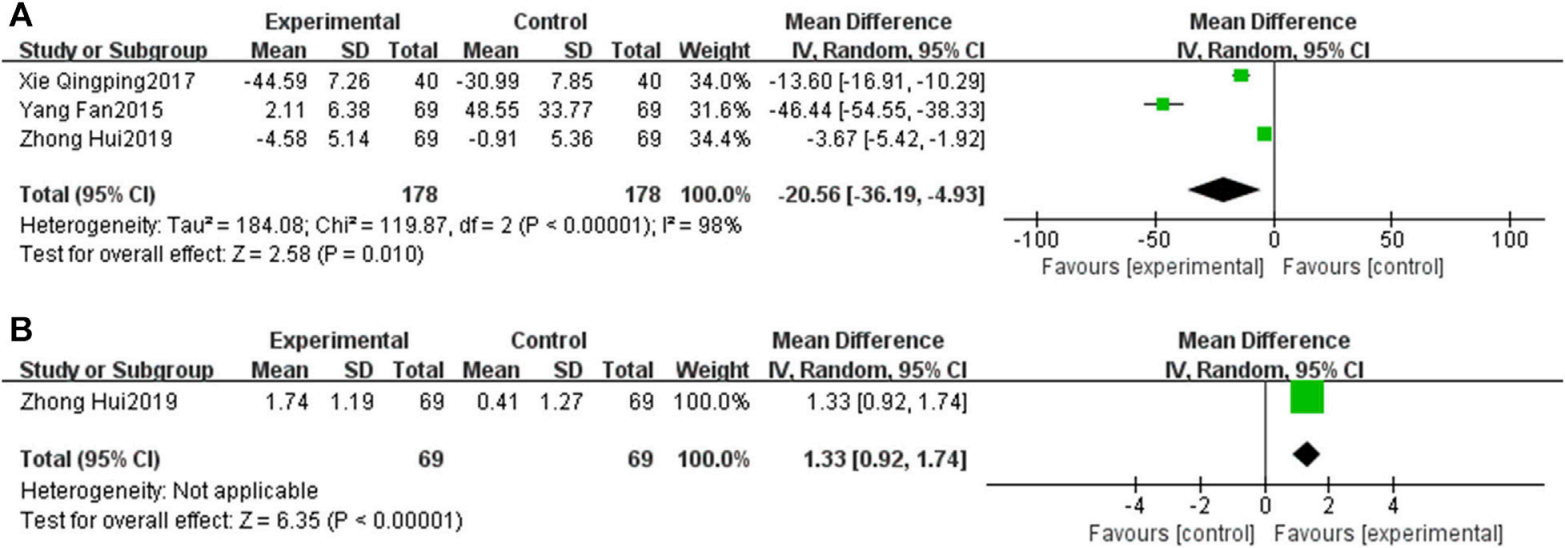
Forest plot of vascular endothelial function [**(A)** ET-1; **(B)** NO].

Three RCTs (Fan et al., 2015; Xie, 2017; Zhong and Zheng, 2019) investigated the ET-1, revealing that ET-1 levels in the EG were significantly reduced compared to those in the CG [MD = −20.56, 95% CI: −36.19 to −4.93, *p* < 0.05; *I* ^2^ = 98%]. Similarly, Zhong and Zheng (2019) reported on NO. The result showed that the NO level in the EG is higher than that in the CG [MD = 1.33, 95% CI: 0.92 to 1.74, *p* < 0.05] (Figure 11).

#### 3.5.7 Oxidative stress

**FIGURE 12:**
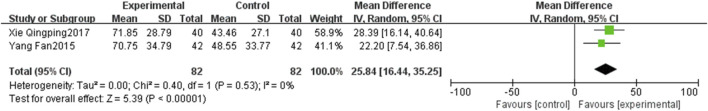
Forest plot of SOD.

Two RCTs (Fan et al., 2015; Xie, 2017) reported on SOD. The results indicated that PNS injection plus CT significantly increases the SOD levels compared with CT alone. [MD = 25.84, 95% CI: 16.44 to 35.52, *p* < 0.05; I^2^ = 0%, random-effects model] (Figure 12).

### 3.6 Safety outcomes

**FIGURE 13:**
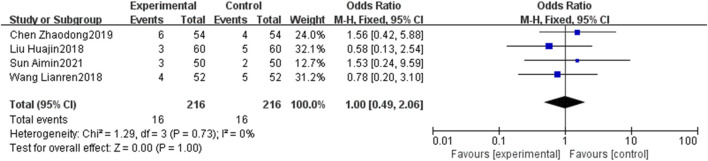
Forest plot of adverse events.

Four RCTs (Liu et al., 2018; Wang, 2018; Chen, 2019; Sun, 2021) assess the adverse effects. Given the non-significant heterogeneity (*p* = 0.73, I^2^ = 0%), a fixed-effects model was employed for the analysis. Figure 13 illustrates that the incidence of adverse events did not significantly differ between the EG and CG [OR = 1.00, 95% CI: 0.49 to 2.06, *p* = 1.00]. Regarding bleeding events, Fan et al. (2015) observed three instances in the CG and one in the EG. Xie (2017) reported two severe bleeding events in the CG, while the EG recorded none. There was no significant difference in the incidence of bleeding events between the two groups (*p* = 0.16).

### 3.7 Subgroup analysis

**FIGURE 14:**
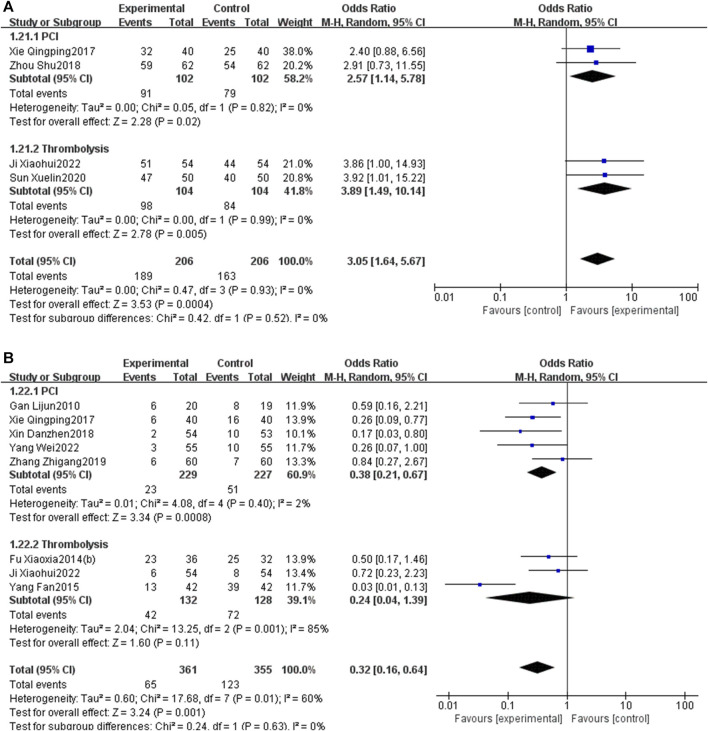
Subgroup analysis stratified by the reperfusion strategies [**(A)** total effective rate; **(B)** MACEs].

Subgroup analysis stratified by the reperfusion strategies (PCI or thrombolysis) indicated that PNS injection could enhance the total effective rate [OR = 2.57, 95% CI: 1.14 to 5.78, *p* < 0.05] and decrease MACEs [OR = 0.38, 95% CI: 0.21 to 0.67, *p* < 0.05] in patients undergoing PCI. Likewise, PNS injection was associated with an increased total effective rate [OR = 3.89, 95% CI: 1.49 to 10.14, *p* < 0.05] and a reduction in MACEs [OR = 0.24, 95% CI: 0.04 to 1.39, *p* < 0.05] in patients who underwent thrombolysis (Figure 14).

**FIGURE 15:**
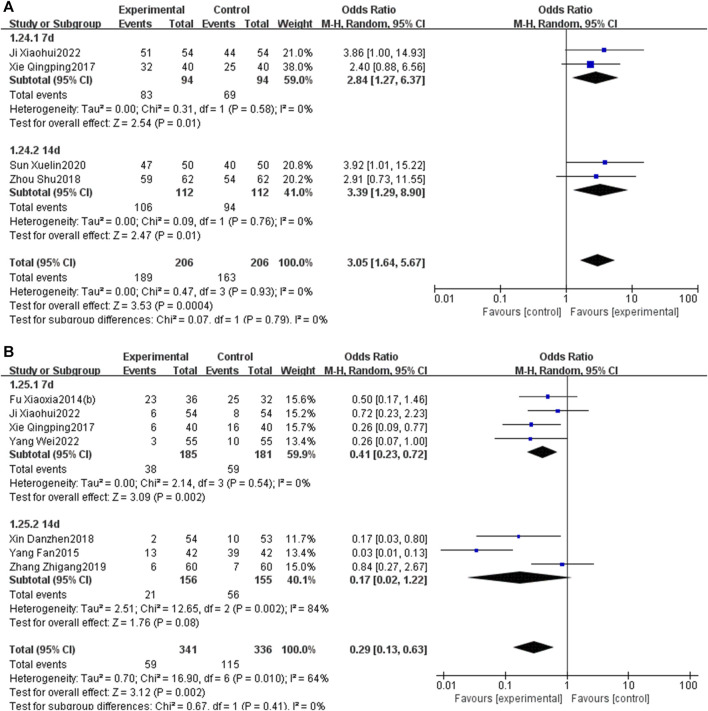
Subgroup analysis stratified by the courses of PNS injection [**(A)** total effective rate; **(B)** MACEs].

The subgroup analysis, stratified by the duration of PNS injection (7 days or 14 days), indicated that a 7-day PNS injection was associated with an increased total effective rate [OR = 2.84, 95% CI: 1.27 to 6.37, *p* < 0.05] and a reduction in MACEs [OR = 0.41, 95% CI: 0.23 to 0.72, *p* < 0.05]. Similarly, a 14-day PNS injection enhanced the total effective rate [OR = 3.39, 95% CI: 1.29 to 8.90, *p* < 0.05] and decreased MACEs [OR = 0.17, 95% CI: 0.02 to 1.22, *p* < 0.05] (Figure 15).

**FIGURE 16:**
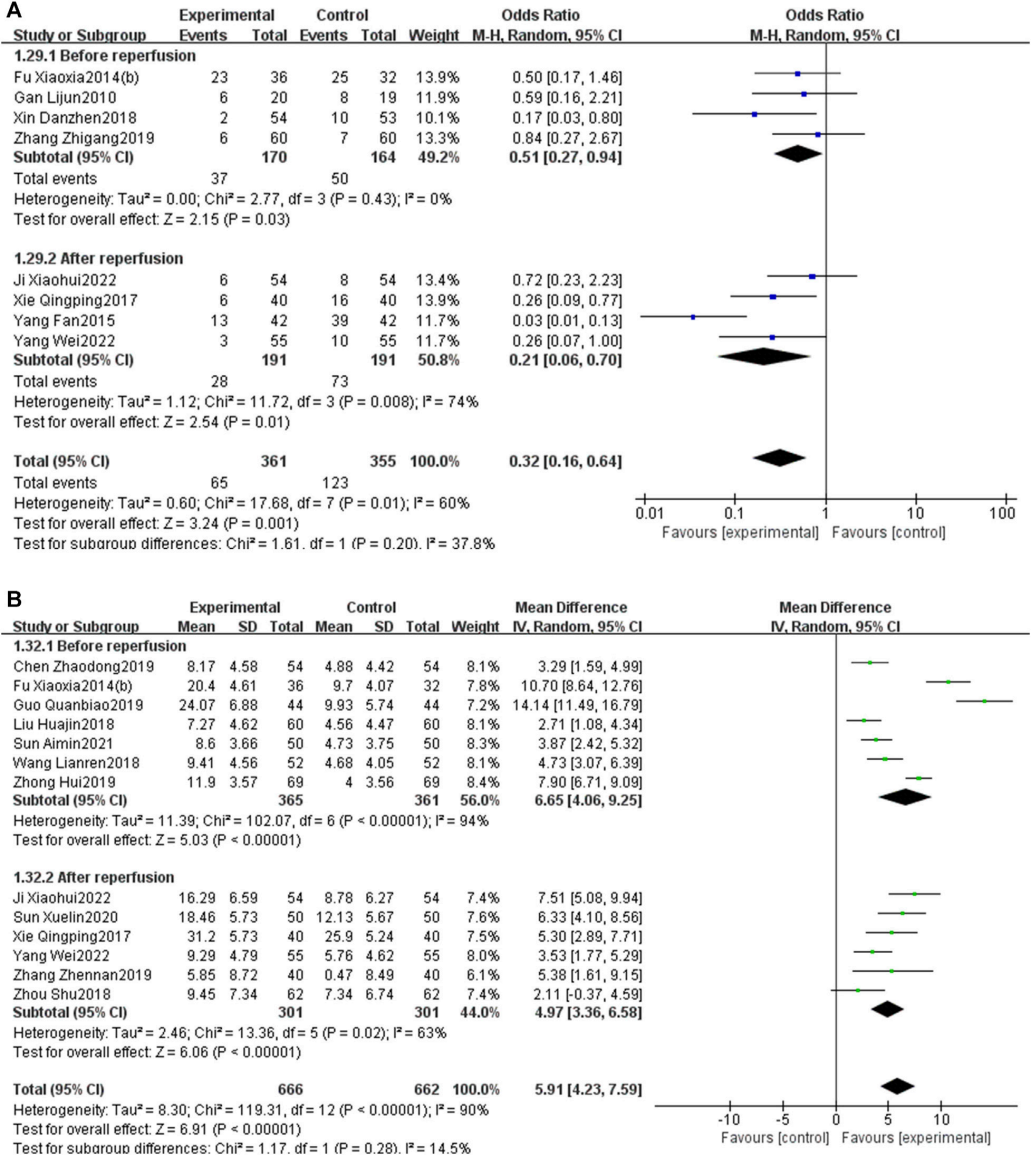
Subgroup analysis stratified by the initial time [**(A)** MACEs; **(B)** LVEF].

Further subgroup analysis, stratified by the initial time of the injection (before reperfusion or after reperfusion), revealed that administering PNS prior to reperfusion was linked to a decrease in MACEs [OR = 0.51, 95% CI: 0.27 to 0.94, *p* < 0.05] and an increase in LVEF [MD = 6.65, 95% CI: 4.06 to 9.25, *p* < 0.05]. Conversely, administering PNS post-reperfusion was also correlated with a reduction in MACEs [OR = 0.21, 95% CI: 0.06 to 0.70, *p* < 0.05] and an elevation in LVEF [MD = 4.97, 95% CI: 3.36 to 6.58, *p* < 0.05] (Figure 16).

**FIGURE 17:**
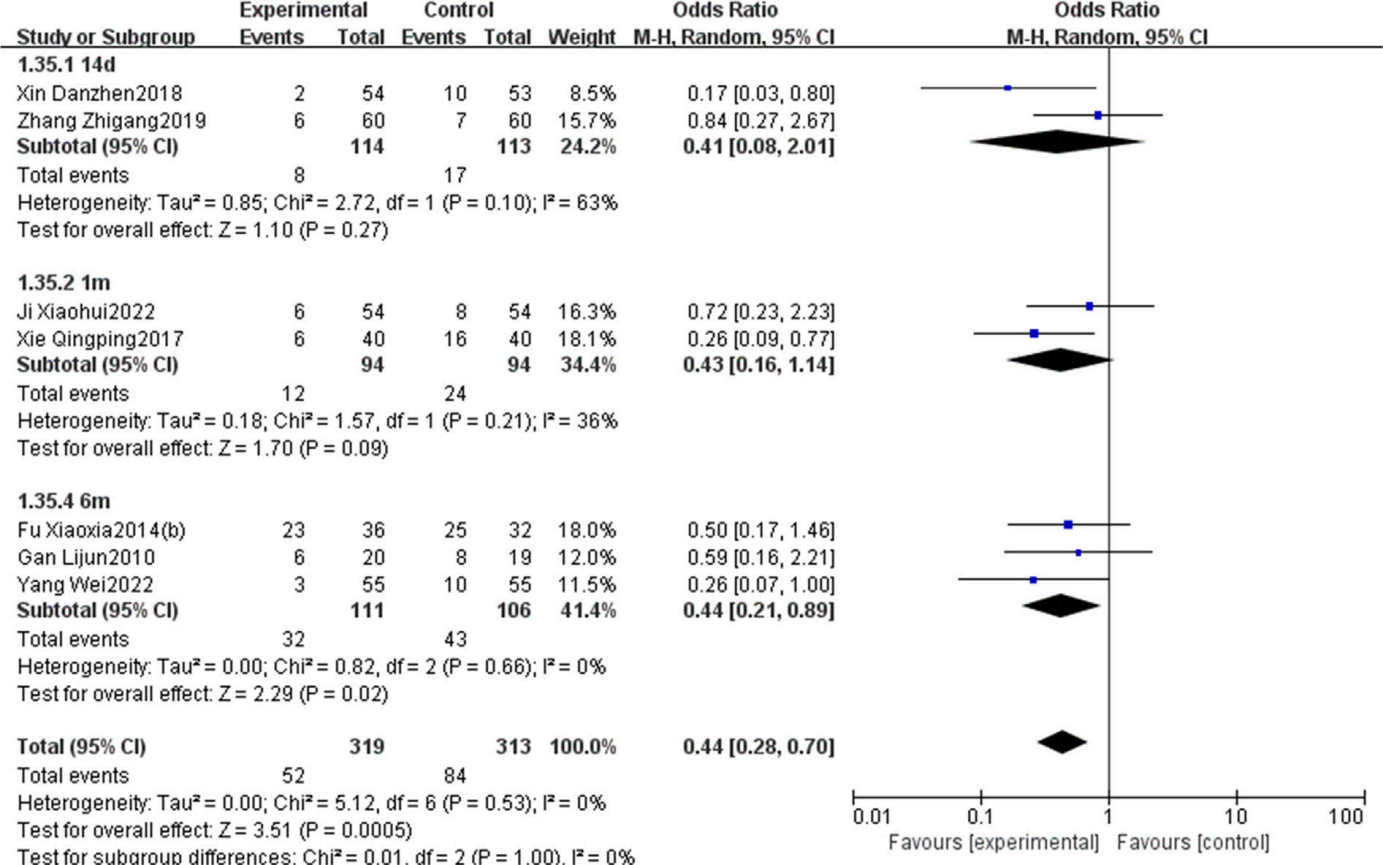
Subgroup analysis stratified by the follow-up time.

An additional subgroup analysis, stratified by the duration of the follow-up, established that the incidence of MACEs in the EG was significantly lower than that in the CG at a 6-month checkpoint [OR = 0.44, 95% CI: 0.21 to 0.89, *p* < 0.05]. However, no significant differences were observed at the 14-day [OR = 0.41, 95% CI: 0.08 to 2.01, *p* = 0.27] and 1-month intervals [OR = 0.43, 95% CI: 0.16 to 1.14, *p* = 0.09] (Figure 17).

### 3.8 Sensitivity analysis

When the heterogeneity was significant, we performed a sensitivity analysis, including MACEs, MIS, CK-MB, cTnT, BNP, LVEF, LVEDD, CRP, TNF-α, and ET-1. Sensitivity analysis found that the reasons may lie in the heterogeneity of trials with respect to the sample size, the age range of participants, PNS dosing protocols, duration of the intervention, and the outcome measures used. After excluding these studies, the heterogeneity significantly reduced, and the results showed a limited impact (Supplementary Table S4). Therefore, these results of meta-analysis were stable and reliable.

### 3.9 Publication bias

**FIGURE 18:**
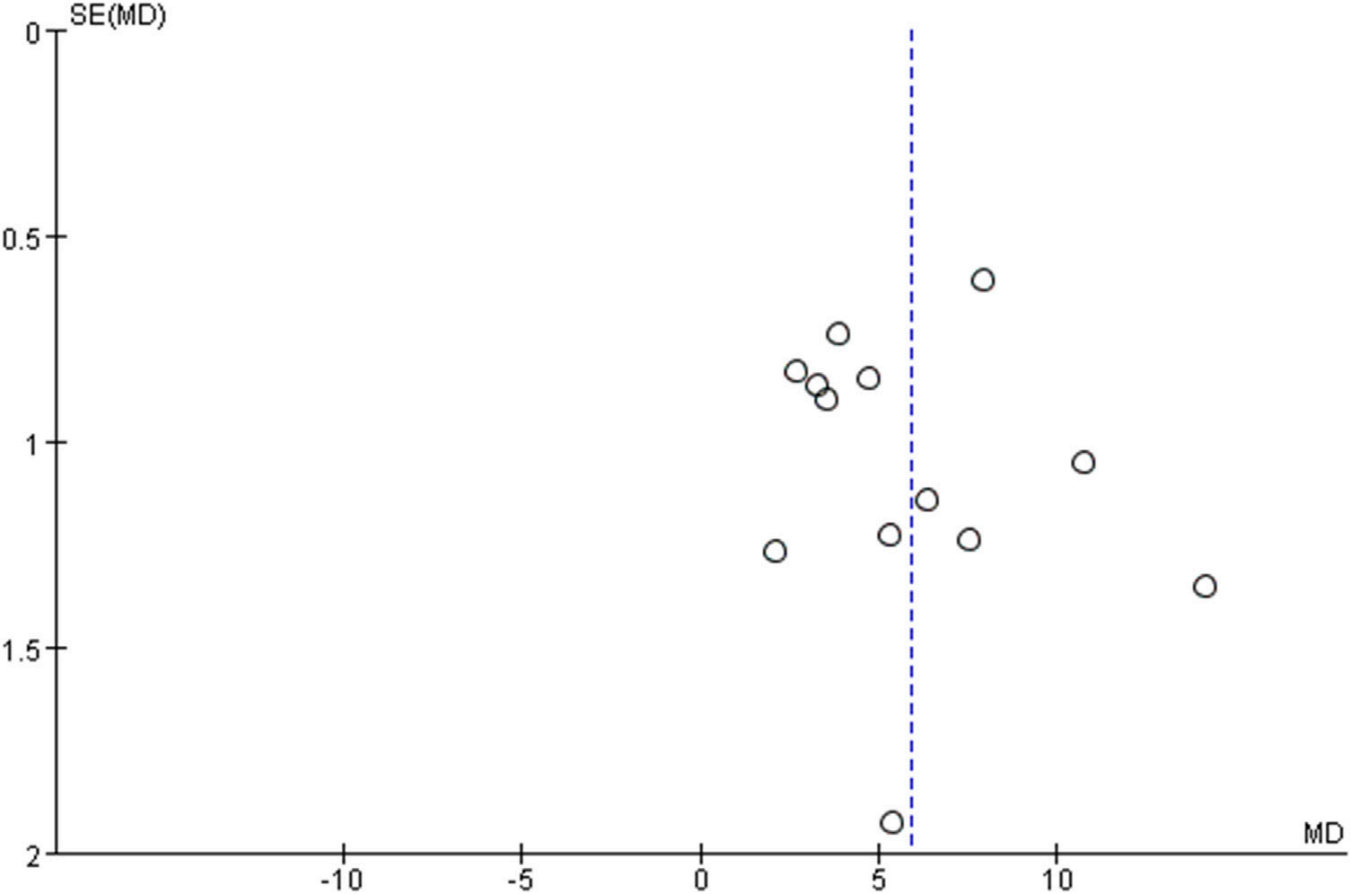
The publication bias of LVEF.

We performed the publication bias for LVEF. As the number of trials was ≥10, we used the funnel chart to determine whether there was publication bias. The results showed that the points of the effects of each trial show a symmetrical inverted funnel distribution, suggesting that the possibility of publication bias was small (Figure 18).

## 4 Discussion

### 4.1 Summary of findings

To the best of our knowledge, this is the first meta-analysis that systematically assesses the efficacy and safety of PNS injection in the treatment of AMI. The findings indicate a significant enhancement in the total effective rate and a reduction in MACEs at 6 months, ST segment elevation, and MIS in AMI patients treated with PNS injection. Additionally, PNS injections were found to improve cardiac and vascular endothelial functions and mitigate myocardial injury, inflammatory responses, and oxidative stress, likely attributing to the protective mechanism of PNS in AMI. Safety assessments suggest that PNS does not increase the incidence of adverse events, particularly bleeding. These results demonstrate the efficacy and safety of PNS injection, offering improved functional outcomes in AMI patients compared to CT alone and potentially presenting a promising treatment modality for AMI. Subgroup analysis further revealed that regardless of the variation in reperfusion strategies (PCI or thrombolysis), initiation timing (pre or post-reperfusion), and duration of treatment (7 or 14 days), PNS exhibited significant therapeutic benefits and favorable long-term prognoses in AMI management. Consequently, PNS is advocated as a secondary treatment in reperfusion AMI patients. The decision to initiate PNS immediately post-reperfusion should consider the risk of bleeding, with a recommended treatment duration of 1–2 weeks.

### 4.2 Mechanism of PNS in AMI

Apoptosis, occurring within hours after the occurrence of AMI, is a primary factor contributing to myocardial injury (Zhou et al., 2018b). This process is intricately linked to the Bcl-2 gene (Bcl-2) and Bcl-2-associated X protein (Bax). PNS is shown to mitigate ultrastructural damage in cardiomyocytes, suppress Bax expression, and enhance the Bcl-2/Bax ratio, thereby reducing cardiomyocyte apoptosis induced by myocardial injury (Zhang et al., 2022). Liu et al. (2019) reported that PNS promotes cardiomyocyte autophagy via the phosphorylation of AMPK and CaMKII, conferring cardioprotection in AMI. Furthermore, PNS has been observed to prevent the decline in mitochondrial membrane potential triggered by hypoxia and serum glucose starvation (SGOD) *in vitro*, inhibiting apoptosis in H9c2 cells. The anti-apoptotic property of PNS may be associated with the upregulation of miR-21 and the inhibition of the PI3K/Akt signaling pathway (Chen et al., 2011; Chen et al., 2019).

Frequent inflammatory reactions aggravate ischemia-reperfusion injury post-AMI, leading to an elevation in MIS (Toldo and Abbate, 2018). TNF-α and IL-6 are the primary inflammatory mediators (Neumann et al., 1995; Frangogiannis, 2014). Fundamental research has demonstrated that PNS can decrease the levels of inflammatory mediators such as nuclear factor kappa B (NF-κB), TNF-α, and IL-6 in rat models of AMI. This reduction mitigates the extent of inflammatory infiltration and myocardial cell edema (Pan et al., 2015; Ning et al., 2020). Sun et al. (2013) and Zhong et al. (2015) corroborated that notoginsenoside R1, a significant phytoestrogen, mitigates H9c2 cell injury by diminishing inflammatory responses and apoptosis in both *in vivo* and *in vitro* experiments.

Oxidative stress injury critically contributes to irreversible and severe damage to cardiomyocytes in AMI (Neri et al., 2015). Experimental findings (Yamada et al., 2019; Wang et al., 2020) confirm that PNS curtails the generation of oxygen-free radicals in cardiomyocytes post-AMI. This modulation alleviates the imbalance between oxidative and antioxidative mechanisms, ameliorating oxidative stress injury and safeguarding the damaged myocardium. Yu et al. (2016) verified through *in vivo* and *in vitro* studies that PNS scavenges oxygen-free radicals, augments the activity of antioxidant enzymes, decreases the expression of endoplasmic reticulum stress-associated proteins, and suppresses the expression of proteins linked to pro-apoptosis.

Alterations in the structure and function of vascular endothelial cells are intimately associated with AMI onset. PNS inhibits the apoptosis of rat vascular endothelial cells by downregulating caspase-3 expression and upregulating the Bcl-2 gene. It enhances endothelium-dependent vasodilation function by elevating NO levels and reducing ET-1 levels (Kwan and Kwan, 2000; Qiao et al., 2015; Zhang et al., 2021). Certain studies (Yang et al., 2016; Zhou et al., 2019) have suggested that angiogenesis induced by PNS post-AMI might correlate with the upregulation of vascular endothelial growth factor A (VEGFA) gene expression. PNS notably promotes the VEGF-stimulated migration of human umbilical vein endothelial cells (HUVECs) and induces approximately a three-fold increase in VEGF mRNA expression.

Platelet activation is a direct precursor to platelet aggregation, potentially leading to AMI. PNS exerts a notable inhibitory effect on platelet-activating factor CD62P and platelet membrane glycoprotein IIb/IIIa. This effect significantly counteracts platelet aggregation and adhesion, enhances fibrinolytic activity, and decelerates thrombosis (Lau et al., 2009). Studies indicate that PNS not only reduces platelet aggregation but also, when used with aspirin, enhances its antiplatelet effect and protects the gastric mucosa. This effect is likely due to PNS’s impact on the metabolic pathways of cyclooxygenase-1 and cytochrome enzymes in the platelet arachidonic acid (AA) pathway (Wang et al., 2021b; Wang et al., 2021c; Dai et al., 2022). Zhong and Zheng (2019) observed that the platelet aggregation rate in the PNS plus CT group was significantly lower than that in the CT group 2 h post-thrombolysis, although no significant difference was noted at 10 h. Although our study did not include indicators related to platelet function and coagulation, future research may address this gap.

### 4.3 Advantages and limitations

The advantages of this meta-analysis included the following. First, a comprehensive search was conducted in a variety of databases without language and time restrictions. Second, independent study selection, data extraction, and bias assessment by two investigators minimized errors. Third, sensitivity analyses did not significantly affect the results, indicating the results were credible. Fourth, multiple subgroup analyses were conducted to assess potential influencing factors and reduce heterogeneity.

There were some limitations in this study. First, the quality of included RCTs was generally low, according to the collaboration tool. None of the 20 RCTs clearly described the method of allocation concealment and blinding, resulting in methodological deficiencies. Therefore, we assessed and reviewed the eligible RCTs by applying considerably high standards. Our meta-analysis attempted to secure methodological rigor and therefore draw unbiased conclusions. Second, all of 20 RCTs were written in Chinese and no English article was involved, and the publication biases could probably affect the results. Third, there were differences in CT of the 20 RCTs, which may increase heterogeneity between the included trials and influence the results. In addition, the heterogeneity in meta-analysis was significant. We used sensitivity and subgroup analyses to explore the source of heterogeneity.

## 5 Conclusion

This study highlighted the efficacy and safety of adjunctive PNS injection in enhancing AMI patient outcomes beyond CT alone. Future RCTs need to solidify these findings through rigorous methods, including double-blinding, enlarged sample sizes, the incorporation of multiple centers, and prolonged follow-up durations.

## Data Availability

The original contributions presented in the study are included in the article/Supplementary Material; further inquiries can be directed to the corresponding authors.
