# Supplementary material for: Efficacy and safety of Panax notoginseng saponin injection in the treatment of acute myocardial infarction: a systematic review and meta-analysis of randomized controlled trials
Source: Front Pharmacol. 2024 Mar 21;15:1353662. doi: 10.3389/fphar.2024.1353662 (PMC10991745; doi:10.3389/fphar.2024.1353662)
Supplement: Supplementary file 1 [file Table1.pdf]

Supplementary Table S1. Full Search Strategy and Results

Database (Search Date)	Search Terms	Filters	Number of results
Pubmed (28/9/2023)	#1 Acute myocardial infarction[mh] OR ST Elevation Myocardial Infarction[mh] OR Myocardial Infarction[mh] OR AMI[tiab] OR STEMI[tiab] OR ST Segment Elevation Myocardial Infarction*[tiab] OR ST Elevated Myocardial Infarction*[tiab]<br>#2 Panax notoginseng saponins*[tiab] OR PNS[tiab] OR Sanqi[tiab] OR Sanchi[tiab] OR Xuesaitong[tiab] OR Xueshuantong[tiab]<br>#3 Randomized controlled trial[mh] OR RCT[tiab]<br>#4 #1 AND #2 AND #3	Language: English	23
Embase (28/9/2023)	#1 (Acute myocardial infarction).exp.<br>#2 (ST Elevation Myocardial Infarction).exp.<br>#3 (Myocardial Infarction).exp.<br>#4 (AMI).ti,ab.<br>#5 (STEMI).ti,ab.<br>#6 (ST Segment Elevation Myocardial Infarction).ti,ab.<br>#7 (ST Elevated Myocardial Infarction).ti,ab.<br>#8 #1 OR #2 OR #3 OR #4 OR #5 OR #6 OR #7<br>#9 (Panax notoginseng saponins).ti,ab.<br>#10 (PNS).ti,ab.<br>#11 (Sanqi).ti,ab.<br>#12 (Sanchi).ti,ab.<br>#13 (Xuesaitong).ti,ab.<br>#14 (Xueshuantong).ti,ab.<br>#15 #9 OR #10 OR #11 OR #12 OR #13 OR #14<br>#16 (Randomized controlled trial).mp.<br>#17 #8 AND #15 AND #16	Language: English	35
Web of science (28/9/2023)	#1 Topic:(Acute myocardial infarction OR ST Elevation Myocardial Infarction OR Myocardial Infarction OR AMI OR STEMI OR ST Segment Elevation Myocardial Infarction* OR ST Elevated Myocardial Infarction*)<br>Databases=SCI-EXPANDED, SSCI, A&HCI, CPCI-S, CPCI-SSH, BKCI-S, BKCI-SSH, ESCI, CCR-EXPANDED, IC Timespan= 1970-2024<br>#2 Topic:(Panax notoginseng saponins* OR PNS OR Sanqi OR Sanchi OR Xuesaitong OR Xueshuantong)<br>Databases=SCI-EXPANDED, SSCI, A&HCI, CPCI-S, CPCI-SSH, BKCI-S, BKCI-SSH, ESCI, CCR-EXPANDED, IC Timespan= 1970-2024<br>#3 Topic:(Randomized controlled trial)<br>Databases=SCI-EXPANDED, SSCI, A&HCI, CPCI-S, CPCI-SSH, BKCI-S, BKCI-SSH, ESCI, CCR-EXPANDED, IC Timespan= 1970-2024<br>#4 #1 AND #2 AND #3	Language: English	21
Cochrane Library (28/9/2023)	“Acute myocardial infarction” OR “ST Elevation Myocardial Infarction” OR “Myocardial Infarction” OR “AMI” OR “STEMI” OR “ST Segment Elevation Myocardial Infarction*” OR “ST Elevated Myocardial Infarction*” in Title Abstract Keyword AND “Panax notoginseng saponins*” OR “PNS” OR	Language: English	12

	“Sanqi” OR “Sanchi” OR “Xuesaitong” OR “Xueshuantong” in Title Abstract Keyword AND “Randomized controlled trial” in Title Abstract Keyword - (Word variations have been searched)		
Chinese National Knowledge Infrastructure (28/9/2023)	("acute myocardial infarction" OR "AMI" OR “st elevated myocardial infarction” OR “STEMI”) AND ("*panax notoginseng saponins*" OR "PNS" OR "sanqi" OR “xuesaitong” OR “xueshuantong”) AND (“randomized controlled trial” OR “RCT”)	Language: Chinese	123
Wanfang (28/9/2023)	("acute myocardial infarction" OR "AMI" OR “st elevated myocardial infarction” OR “STEMI”) AND ("*panax notoginseng saponins*" OR "PNS" OR "sanqi" OR “xuesaitong” OR “xueshuantong”) AND (“randomized controlled trial” OR “RCT”)	Language: Chinese	112
VIP (28/9/2023)	("acute myocardial infarction" OR "AMI" OR “st elevated myocardial infarction” OR “STEMI”) AND ("*panax notoginseng saponins*" OR "PNS" OR "sanqi" OR “xuesaitong” OR “xueshuantong”) AND (“randomized controlled trial” OR “RCT”)	Language: Chinese	80

Supplementary Table S2. The Exclusion Studies and Reasons

Reason for Exclusion	Study Name	Reference
inaccurate data	Mu XH et al 2021	Clinical observation on 45 cases of acute myocardial infarction treated by thrombolysis with urokinase combined with Xuesaitong. Chinese Community Physician, 2013,24:73-74.
inaccurate data	Rong SS et al 2002	Clinical observation of thrombolytic therapy combined with Xuesaitong in the treatment of senile acute myocardial infarction [J]. Shanghai Journal of traditional Chinese Medicine, 2002,07:11-12.
inaccurate data	Dai CB et al 2020	Clinical observation of Xuesaitong injection combined with low molecular weight heparin in the treatment of non-ST segment elevation myocardial infarction [J]. Chinese Medical Sciences, 2011,11:52+54.
ineligible interventions	Li H et al 2020	Clinical observation of compound Xueshuantong capsule in the treatment of acute myocardial infarction of coronary heart disease [J]. Emergency of traditional Chinese Medicine, 2020,10:1841-1843.
ineligible interventions	Tong LJ et al 2022	Clinical study of Xuesaitong granule combined with rosuvastatin in the treatment of anterior wall myocardial infarction after PCI. Jiangxi Medicine, 2022,07:756-758+762.
ineligible interventions	Fang JZ et al 2010	Effect of Xuesaitong capsule on cardiac remodeling and serum procollagen III level in patients with acute myocardial infarction [J]. Medical Herald, 2010,11:1458-1459.
ineligible interventions	Wu D et al 2015	Effect of Xueshuantong capsule on acute myocardial ischemia and antithrombosis [J]. Chinese Journal of Comparative Medicine, 2015,12:10-14+25.
ineligible interventions	Nan Ma et al 2020	Intervention effect of compound Xueshuantong capsule combined with nicorandil on no reflow after PCI in elderly patients with ST segment elevation acute myocardial infarction. Clinical Medical Research and practice,
non-RCT study design	Wang W et al 2011	Combination of Panax notoginseng saponins and aspirin potentiates platelet inhibition with alleviated gastric injury via modulating arachidonic acid metabolism. Biomed Pharmacother. 2021;134:111165.
non-RCT study design	Wang D 2021	Cardioprotection of Panax Notoginseng saponins against acute myocardial infarction and heart failure through inducing autophagy. Biomed Pharmacother. 2021;136:111287.
non-RCT study design	Yang BR et al 2016	Amelioration of acute myocardial infarction by saponins from flower buds of Panax notoginseng via pro-angiogenesis and anti-apoptosis. J Ethnopharmacol. 2016;181:50-8.
non-RCT study design	Liu XW et al 2019	Panax Notoginseng Saponins Attenuate Myocardial Ischemia-Reperfusion Injury Through the HIF-1α/BNIP3 Pathway of Autophagy. J Cardiovasc Pharmacol. 2019;73(2):92-99.
non-RCT study design	Fang M et al 2022	The Synergistic Mechanism of Total Saponins and Flavonoids in Notoginseng-Safflower against Myocardial Infarction Using a Comprehensive Metabolomics Strategy. Molecules. 2022 ;27(24):8860.

non-RCT study design	Rong WQ et al 2023	Research progress on the preventive and therapeutic effect of Panax notoginseng on acute myocardial infarction [J]. Journal of traditional Chinese Medicine, 2023,04: 104-107.
non-RCT study design	Li Jl et al 2013	Protective effects of Panax notoginseng saponins on acute myocardial infarction in rats [J]. Pharmacology and Clinic of traditional Chinese Medicine, 2013,01:68-72.
non-RCT study design	Jiang BJ et al 2018	Clinical effect of Panax notoginseng in the treatment of cardiovascular disease [J]. Electronic Journal of Cardiovascular Diseases of Integrated traditional Chinese and Western Medicine, 2018,13:163+166.
non-RCT study design	Wei LL et al 2023	Meta-analysis and network pharmacological study on the efficacy of ginseng-Panax notoginseng in the treatment of myocardial infarction based on Shenqi decoction [J]. World traditional Chinese Medicine, 2023,14:2030-2039.

Supplementary Table S3. The composition of the prescriptions

Study	Formulation	Source	Compound, concentration	Quality control reported? (Y/N)	Chemical analysis reported? (Y/N)
Yang Wei2022	Xueshuantong injection (lyophilized)	Guangxi Wuzhou Pharmaceutical Co., Ltd	PNS, 150mg	Y – Prepared according to National Drug Standards of China Food and Drug Administration (Z20025652)	N
Yang Fan2015	Xueshuantong injection (lyophilized)	Guangxi Wuzhou Pharmaceutical Co., Ltd	PNS, 250mg	Y – Prepared according to National Drug Standards of China Food and Drug Administration (Z20025652)	N
Zhong Hui2019	Xueshuantong injection (lyophilized)	Guangxi Wuzhou Pharmaceutical Co., Ltd	PNS, 500mg	Y – Prepared according to National Drug Standards of China Food and Drug Administration (Z20025652)	N
Xie Qingping2017	Xueshuantong injection (lyophilized)	Guangxi Wuzhou Pharmaceutical Co., Ltd	PNS, 150mg	Y – Prepared according to National Drug Standards of China Food and Drug Administration (Z20025652)	N
Guo Quanbiao2019	Xuesaitong injection	Heilongjiang Zhenbaodao pharmaceutical Co., Ltd	PNS, 100mg	Y – Prepared according to National Drug Standards of China Food and Drug Administration (Z23020787)	N
Sun Aimin2021	Xuesaitong injection	Jiangsu Langou pharmaceutical Co., Ltd	PNS, 100mg	Y – Prepared according to National Drug Standards of China Food and Drug Administration (Z32020418)	N
Zhou Shu2018	Xuesaitong injection	Heilongjiang Zhenbaodao pharmaceutical Co., Ltd	PNS, 100mg	Y – Prepared according to National Drug Standards of China Food and Drug Administration (Z23020787)	N

Zhang Zhigang2019	Xuesaitong injection	Xian Libang pharmaceutical Co., Ltd	PNS, 100mg	Y – Prepared according to National Drug Standards of China Food and Drug Administration (Z61021575)	N
Chen Zhaodong2019	Xuesaitong injection	Hubei Tiansheng pharmaceutical Co., Ltd	PNS, 100mg	Y – Prepared according to National Drug Standards of China Food and Drug Administration (Z42020390)	N
Qiao Zhili2016	Xuesaitong injection	Heilongjiang Zhenbaodao pharmaceutical Co., Ltd	PNS, 100mg	Y – Prepared according to National Drug Standards of China Food and Drug Administration (Z23020787)	N
Sun Xuelin2020	Xuesaitong injection (lyophilized)	Kunyao pharmaceutical Co., Ltd	PNS, 200mg	Y – Prepared according to National Drug Standards of China Food and Drug Administration (Z20026438)	N
Ji Xiaohui2022	Xueshuantong injection (lyophilized)	Guangxi Wuzhou Pharmaceutical Co., Ltd	PNS, 150mg	Y – Prepared according to National Drug Standards of China Food and Drug Administration (Z20025652)	N
Xin Danzhen2018	Xuesaitong injection	Yunnan Baiyao pharmaceutical Co., Ltd	PNS, 100mg	Y – Prepared according to National Drug Standards of China Food and Drug Administration (Z53021499)	N
Zhang Zhennan2019	Xuesaitong injection	Anhui Shengying pharmaceutical Co., Ltd	PNS, 100mg	Y – Prepared according to National Drug Standards of China Food and Drug Administration (Z20143024)	N
Fu Xiaoxia2014（a）	Xueshuantong injection (lyophilized)	Guangxi Wuzhou Pharmaceutical Co., Ltd	PNS, 250mg	Y – Prepared according to National Drug Standards of China Food and Drug Administration (Z20025652)	N

Fu Xiaoxia2014（b）	Xueshuantong injection (lyophilized)	Guangxi Wuzhou Pharmaceutical Co., Ltd	PNS, 250mg	Y – Prepared according to National Drug Standards of China Food and Drug Administration (Z20025652)	N
Liu Huajin2018	Xuesaitong injection (lyophilized)	Xianan Hanfeng pharmaceutical Co., Ltd	PNS, 100mg	Y – Prepared according to National Drug Standards of China Food and Drug Administration (Z61021576)	N
Wang Lianren2018	Xuesaitong injection	Hubei Tiansheng pharmaceutical Co., Ltd	PNS, 100mg	Y – Prepared according to National Drug Standards of China Food and Drug Administration (Z42020390)	N
Feng Guanpeng2017	Xuesaitong injection (lyophilized)	Haerbin Zhenbao Pharmaceutical Co., Ltd	PNS, 200mg	Y – Prepared according to National Drug Standards of China Food and Drug Administration (Z20026437)	N
Gan Lijun2010	Xuesaitong injection	Kunming Xingzhong pharmaceutical Co., Ltd	PNS, 100mg	Y – Prepared according to National Drug Standards of China Food and Drug Administration (Z53021499)	N

Supplementary Table S4. The results of sensitivity analysis

Outcome	Study removed [first author(year)]	P-value, I^2-value	P-value	OR/MD [95% CI]
MIS	Sun Xuelin2020	P=0.86 I^2=0%	P<0.05	-7.65[-8.62,-6.68]
CK-MB	Xie Qingping2017	P=0.24 I^2=27%	P＞0.05	-42.06[-85.62,1.50]
cTnT	Yang Fan2015	P=0.45 I^2=0%	P<0.05	-13.46[-18.16,-8.75]
BNP	Fu Xiaoxia2014（a）	P=0.86 I^2=0%	P<0.05	-73.85[-96.08,-51.63]
TNF-α	Zhou Shu2018	P=1.00 I^2=0%	P<0.05	-7.26[-8.57,-5.96]
MACEs	Yang Fan2015	P=0.53 I^2=0%	P<0.05	0.44[0.28,0.70]

Supplementary Table S5. The PRISMA 2020 Checklist

Section and Topic	Item #	Checklist item	Location where item is reported
**TITLE**			
Title	1	Identify the report as a systematic review.	
**ABSTRACT**			
Abstract	2	See the PRISMA 2020 for Abstracts checklist.	
**INTRODUCTION**			
Rationale	3	Describe the rationale for the review in the context of existing knowledge.	
Objectives	4	Provide an explicit statement of the objective(s) or question(s) the review addresses.	
**METHODS**			
Eligibility criteria	5	Specify the inclusion and exclusion criteria for the review and how studies were grouped for the syntheses.	
Information sources	6	Specify all databases, registers, websites, organisations, reference lists and other sources searched or consulted to identify studies. Specify the date when each source was last searched or consulted.	
Search strategy	7	Present the full search strategies for all databases, registers and websites, including any filters and limits used.	
Selection process	8	Specify the methods used to decide whether a study met the inclusion criteria of the review, including how many reviewers screened each record and each report retrieved, whether they worked independently, and if applicable, details of automation tools used in the process.	
Data collection process	9	Specify the methods used to collect data from reports, including how many reviewers collected data from each report, whether they worked independently, any processes for obtaining or confirming data from study investigators, and if applicable, details of automation tools used in the process.	
Data items	10a	List and define all outcomes for which data were sought. Specify whether all results that were compatible with each outcome domain in each study were sought (e.g. for all measures, time points, analyses), and if not, the methods used to decide which results to collect.	
	10b	List and define all other variables for which data were sought (e.g. participant and intervention characteristics, funding sources). Describe any assumptions made about	

Section and Topic	Item #	Checklist item	Location where item is reported
		any missing or unclear information.	
Study risk of bias assessment	11	Specify the methods used to assess risk of bias in the included studies, including details of the tool(s) used, how many reviewers assessed each study and whether they worked independently, and if applicable, details of automation tools used in the process.	
Effect measures	12	Specify for each outcome the effect measure(s) (e.g. risk ratio, mean difference) used in the synthesis or presentation of results.	
Synthesis methods	13a	Describe the processes used to decide which studies were eligible for each synthesis (e.g. tabulating the study intervention characteristics and comparing against the planned groups for each synthesis (item #5)).	
	13b	Describe any methods required to prepare the data for presentation or synthesis, such as handling of missing summary statistics, or data conversions.	
	13c	Describe any methods used to tabulate or visually display results of individual studies and syntheses.	
	13d	Describe any methods used to synthesize results and provide a rationale for the choice(s). If meta-analysis was performed, describe the model(s), method(s) to identify the presence and extent of statistical heterogeneity, and software package(s) used.	
	13e	Describe any methods used to explore possible causes of heterogeneity among study results (e.g. subgroup analysis, meta-regression).	
	13f	Describe any sensitivity analyses conducted to assess robustness of the synthesized results.	
Reporting bias assessment	14	Describe any methods used to assess risk of bias due to missing results in a synthesis (arising from reporting biases).	
Certainty assessment	15	Describe any methods used to assess certainty (or confidence) in the body of evidence for an outcome.	
**RESULTS**			
Study selection	16a	Describe the results of the search and selection process, from the number of records identified in the search to the number of studies included in the review, ideally using a flow diagram.	

Section and Topic	Item #	Checklist item	Location where item is reported
	16b	Cite studies that might appear to meet the inclusion criteria, but which were excluded, and explain why they were excluded.	
Study characteristics	17	Cite each included study and present its characteristics.	
Risk of bias in studies	18	Present assessments of risk of bias for each included study.	
Results of individual studies	19	For all outcomes, present, for each study: (a) summary statistics for each group (where appropriate) and (b) an effect estimate and its precision (e.g. confidence/credible interval), ideally using structured tables or plots.	
Results of syntheses	20a	For each synthesis, briefly summarise the characteristics and risk of bias among contributing studies.	
	20b	Present results of all statistical syntheses conducted. If meta-analysis was done, present for each the summary estimate and its precision (e.g. confidence/credible interval) and measures of statistical heterogeneity. If comparing groups, describe the direction of the effect.	
	20c	Present results of all investigations of possible causes of heterogeneity among study results.	
	20d	Present results of all sensitivity analyses conducted to assess the robustness of the synthesized results.	
Reporting biases	21	Present assessments of risk of bias due to missing results (arising from reporting biases) for each synthesis assessed.	
Certainty of evidence	22	Present assessments of certainty (or confidence) in the body of evidence for each outcome assessed.	
**DISCUSSION**			
Discussion	23a	Provide a general interpretation of the results in the context of other evidence.	
	23b	Discuss any limitations of the evidence included in the review.	
	23c	Discuss any limitations of the review processes used.	
	23d	Discuss implications of the results for practice, policy, and future research.	
**OTHER INFORMATION**			
Registration and	24a	Provide registration information for the review, including register name and registration number, or state that the	

Section and Topic	Item #	Checklist item	Location where item is reported
protocol		review was not registered.	
	24b	Indicate where the review protocol can be accessed, or state that a protocol was not prepared.	
	24c	Describe and explain any amendments to information provided at registration or in the protocol.	
Support	25	Describe sources of financial or non-financial support for the review, and the role of the funders or sponsors in the review.	
Competing interests	26	Declare any competing interests of review authors.	
Availability of data, code and other materials	27	Report which of the following are publicly available and where they can be found: template data collection forms; data extracted from included studies; data used for all analyses; analytic code; any other materials used in the review.	
